# Novel All-Nitrogen Molecular Crystals Composed of Tetragonal N_4_ Molecules

**DOI:** 10.3390/ijms23105503

**Published:** 2022-05-14

**Authors:** Suna Pang, Feng Wang

**Affiliations:** School of Physics, Beijing Institute of Technology, Beijing 100081, China; 3120195765@bit.edu.cn

**Keywords:** high-energy density material, detonation pressures, detonation velocities, thermodynamic stability

## Abstract

A computational study promises insight into molecular crystals consisting of the tetrahedral form of N${}_{4}$ molecules (Td-N${}_{4}$). Here, our efforts are focused on theoretically predicting the existence of the molecular crystals consisting of Td-N${}_{4}$ molecules. On the basis of the first principles of Born–Oppenheimer molecular dynamics under constant temperature and pressure, and geometry optimizations under hydrostatic pressures without any constrained parameters, molecular crystals consisting of Td-N${}_{4}$ molecules were confirmed to be dynamically and thermally metastable. Our analysis shows that, with high detonation performance and high stability, these Td-N${}_{4}$ molecular crystals can indeed be potential candidates as high-energy density explosives.

## 1. Introduction

There is currently much interest in studying all-nitrogen molecules motivated by their considerable potential for applications as high-energy density materials (HEDMs). While ongoing theoretical publications predicted many species of all-nitrogen molecules and ions as possible HEDMs, such as N${}_{3}$, N${}_{4}$, N${}_{5}^{+}$, N${}_{5}^{-}$, N${}_{6}$, N${}_{8}$, N${}_{10}$ and even N${}_{60}$, only a handful of these were synthesized and experimentally detected [1,2,3,4,5,6,7,8,9,10,11,12,13,14,15,16,17]. There is still a lack of a suitable universal method for the synthesis of all-nitrogen compounds because of their high endothermicity. A large number of theoretical studies on all-nitrogen molecules and ions are crucial in exploring the range and conditions of their existence. The release of energy is a direct reason for studying all-nitrogen compounds as HEDMs. The energy of all-nitrogen compounds comes from bond dissociation energy, significantly varying from the single bond to double and triple bonds. The bond dissociation enthalpy of the N≡N triple bond is 228 kcal/mol, which renders it a very stable chemical bond [18]. Therefore, the conversion of all-nitrogen compounds into N${}_{2}$ molecules is accompanied by a large amount of energy release. In addition, all-nitrogen compounds are clean materials because their decomposition only produces environmentally friendly nitrogen (N${}_{2}$) without harmful waste.

Experimentally, there have been advances in the synthesis of some neutral and charged all-nitrogen species, such as N${}_{3}$, N${}_{3}^{+}$, N${}_{3}^{-}$, N${}_{4}$, N${}_{5}^{+}$, and N${}_{5}^{-}$ [1,4,5,6,7,10]. The above-listed all-nitrogen species are unfortunately all short-lived intermediates whose thermodynamic stability is rather insignificant to be used in practice, although they play important roles in atmospheric chemistry [19,20]. Theoretically, there is continuing interest in all-nitrogen molecular crystals. For instance, Hirshberg et al. predicted that a molecular solid consisting of N${}_{8}$ molecules is metastable under ambient pressure. The interaction between N${}_{8}$ molecules is van der Waals force and electrostatic force. The authors above mainly discussed the crystal structure and performance of N${}_{8}$ molecule crystals [21]. Subsequently, Michael et al. showed that N${}_{6}$ crystals are dynamically metastable at room temperature by metadynamic simulations, but dissociate to N${}_{2}$ molecules above 700 K [22]. Metastable all-nitrogen molecular crystals consisting of N${}_{10}$ molecules were recently reported by Shijie Liu et al. [23] The structures of these molecular crystals are affected by external pressure, and their decomposition temperature is much higher than room temperature. There is experimental evidence for the isolation of Td-N${}_{4}$ [18], although the conclusive assignment of spectral features requires further experimentation. In addition, several theoretical studies [24,25,26] showed that Td-N${}_{4}$ is a metastable species that would have a reasonably long lifetime. Correspondingly, inspired by the above studies, some questions related to Td-N${}_{4}$ arise: (1) What are the structural and electronic properties of the molecular crystals of Td-N${}_{4}$ under hydrostatic pressures? (2) Are the molecular crystals of Td-N${}_{4}$ stable under ambient temperature and pressure? To answer these and other related questions requires further theoretical and experimental analysis of the system.

To the best of our knowledge, the stability of useful energetic material must be appropriate. On the one hand, it needs to easily react with other substances to release a large amount of energy. On the other hand, it must be stable enough to be synthesized and stored without spontaneous decomposition. In this paper, of particular interest to us was to study the structure and stability of molecular crystals of Td-N${}_{4}$ on the basis of Born–Oppenheimer molecular dynamics (BO-MD) and geometry optimization under hydrostatic pressure without any constrained parameters.

This paper is organized as follows. In Section 2, we briefly introduce the theoretical methods. In Section 3, we analyze the calculation results, and the section is divided into three parts. First, we report the pressure-induced changes of lattice constants along three lattice orientations. The ratio of lattice constants at different pressure levels is calculated for these crystals. Next, to understand the stability of the crystal structures, we calculated their electron structure and density of states (DOS), along with molecular dynamics (MD) simulations using a supercell consisting of 8 Td-N${}_{4}$ molecules. Lastly, detonation velocity D and detonation pressure P were predicted using the program that we had written. Conclusions are drawn in Section 4.

## 2. Computational Details

### 2.1. Method

In this section, the theoretical methods used in this study are briefly summarized. Our calculations were all performed with the PWmat package (PWmat is a plane wave pseudopotential package for density functional theory calculations) [27,28] using the norm-conserving pseudopotential. The PWmat package is implemented with the graphics processing unit (GPU) and could speed up calculations by 20 times compared to the central processing unit (CPU). The optimized geometry and electronic structure are studied using density-functional theory (DFT). The exchange correlation functional is described by Perdew, Burke, and Ernzerhof with generalized gradient approximation (GGA-PBE) [29].

For the method of the MD algorithm, constant-temperature and constant-pressure Langevin dynamics [30,31] was applied. The structure was relaxed prior to MD simulations. For more accurate calculations, the norm-conserving pseudopotential was chosen, and the PBE was implemented to deal with exchange correlation interaction. The cutoff energy was set to be 80 Ry (1 Ry = 13.6 eV), which is high enough to ensure the convergence of total energy and stress. MD simulations were performed at 500 K under 1 and 10 GPa pressure. The time step for the MD simulations was 1 fs. The convergence accuracy between two self-consistent iterations was chosen to be 2 × 10${}^{-5}$ eV, and maximal atomic force of 0.02 eV/$\overset{˚}{A}$ was used as a convergence criterion for all optimizations with and without hydrostatic pressure. For the pressurization algorithm, geometry optimizations under hydrostatic pressure without any constrained parameters were performed using the conjugated gradient algorithm with a convergence criterion of 1 × 10${}^{-4}$ a.u. on displacement and 2 × 10${}^{-5}$ eV on energy. To assist with the convergence comparison, we used an equivalent Monkhorst–Pack k-point mesh for different supercell sizes. The Monkhorst–Pack k-point of 4 × 4 × 4 was used to sample the Brillouin zone in all structural optimizations and other relevant computations.

### 2.2. Characterization of Atomic Structure

The evolution of the Td-N${}_{4}$ molecular crystal structure with pressure was analyzed with the pair distribution function (PDF). The PDF counts atomic pairs at distance *r*.

In practice, one takes each atom as the origin of coordinates, the number of atoms (*N*) within the spherical shell r∼r+Δr is counted, and the average number of atoms 〈N〉 at distance *r* is obtained. A probability distribution can be defined as follows:

(1) $$G(r)=\frac{\langle N\rangle }{4\pi r^{2}\Delta r}$$

**Figure 1:**
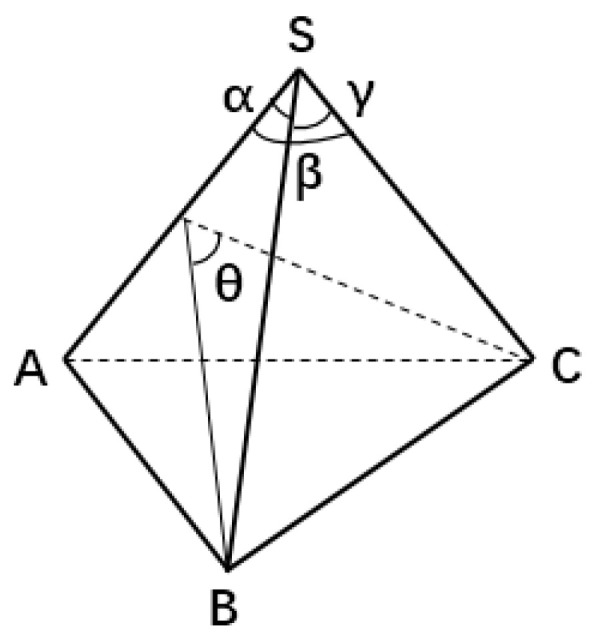
Schematic diagram of trihedral angle S-ABC; α,β,γ are three face angles; θ is the dihedral between two planes △SAB and △SAC.

Similarly, a trihedral angular distribution function (ADF) and a dihedral distribution function (DDF) can be defined. The ADF is the statistic within which a trihedral angle is composed of three atoms within a certain radius, while the DDF is the statistic within which a dihedral angle in a tetrahedron is composed of four atoms within a certain radius. As shown in Figure 1, the edges formed the face angles of the trihedral angle, α,β,γ, and the faces formed the dihedral angles of the trihedral angle, such as θ; they are related as follows:

(2) $$\cos\theta =\frac{\cos\gamma -\cos\alpha \cos\beta }{\sin\alpha \sin\beta }$$

## 3. Results and Discussion

### 3.1. Properties under Hydrostatic Pressures

**Figure 2:**
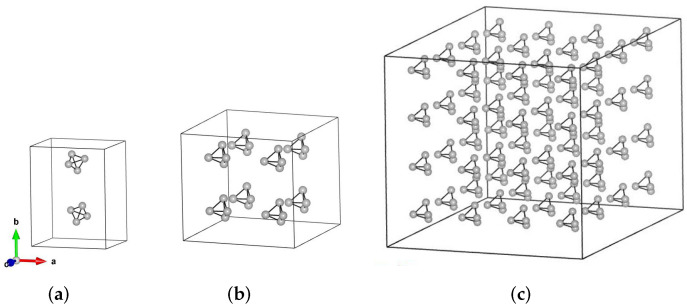
Crystal structures, with little gray balls representing nitrogen atoms. (**a**) This crystal contains 2 Td-N${}_{4}$ molecules, lattice parameters are a = 6.58 $\overset{˚}{A}$, b = 8.52 $\overset{˚}{A}$, c = 6.60 $\overset{˚}{A}$, α = 92.6${}^{\circ }$, β = 89.3${}^{\circ }$, γ = 92${}^{\circ }$. (**b**) This crystal contains 8 Td-N${}_{4}$ molecules; lattice parameters are a = 8.21 $\overset{˚}{A}$, b = 8.36 $\overset{˚}{A}$, c = 8.02 $\overset{˚}{A}$, α = 116${}^{\circ }$, β = 117${}^{\circ }$, γ = 96${}^{\circ }$. (**c**) This crystal contains 64 Td-N${}_{4}$ molecules; lattice parameters are a = 16.42 $\overset{˚}{A}$, b = 16.72 $\overset{˚}{A}$, c = 16.04 $\overset{˚}{A}$, α = 116${}^{\circ }$, β = 117${}^{\circ }$, γ = 96${}^{\circ }$.

In our work, we constructed and optimized a series of Td-N${}_{4}$ molecular crystals. These crystal structures were composed of 2, 8, and 64 Td-N${}_{4}$ molecules and were lower in energy, so they are more stable than other crystals. Therefore, we chose these crystal structures to study, as shown in Figure 2. For convenience, they are denoted as 2-Td-N${}_{4}$, 8-Td-N${}_{4}$, and 64-Td-N${}_{4}$, respectively. To stabilize molecular crystals on a macroscopic scale, the internal molecular structure should be stable in the gas phase. First, the geometry of Td-N${}_{4}$ is optimized at the PBE level of theory, and the geometry was optimized with PWmat. The bond length between N–N atoms of Td-N${}_{4}$ was around 1.47 $\overset{˚}{A}$, which is consistent with the previous report of 1.458 $\overset{˚}{A}$ by Bittererovás et al. [32] Results show that this structure could be regarded to be a standard Td-N${}_{4}$ because the symmetry was incomparably high.

**Figure 3:**
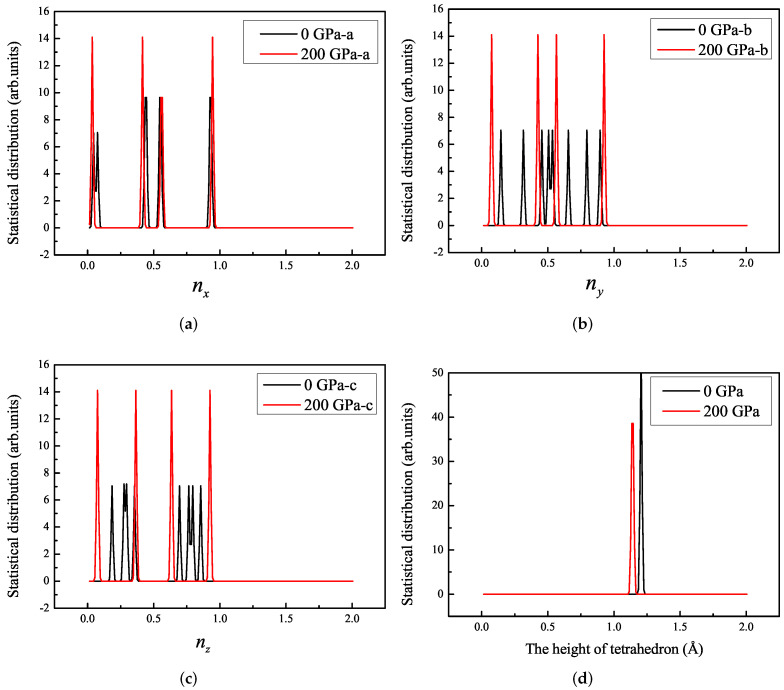
Statistical distribution of components of the unit vector in the normal direction on the surface of tetrahedron N${}_{4}$ for 2-Td-N${}_{4}$ under 0 GPa and 200 GPa: (**a**) $n_{x}$, (**b**) $n_{y}$, (**c**) $n_{z}$; (**d**) statistical distribution of the heights of the tetrahedron N${}_{4}$.

In order to intuitively understand the arrangement or mutual orientation of individual tetrahedra in a crystal, we wrote a program (statistical distribution of the components of the unit vector in the normal direction on the surface of tetrahedron N${}_{4}$) to count the direction of the normal plane and the distance from each vertex to the plane, such as the distance from vertex S to plane △ABC, from vertex A to plane △SBC, from vertex B to plane △SAC, and from vertex C to plane △SAB, as shown in the Figure 1. We obtained the components of each normal plane in three directions and the distance from each vertex to the plane. The abscissa of Figure 3a–c is the three components of our defined direction vector $\vec{n}=\left(n_{x},n_{y},n_{z}\right)$. As shown in Figure 3a–c, the solid black line represents the optimized structure without pressure, which comprises multiple peaks in each component, indicating that the mutual orientation of the tetrahedrons in the crystal cell was irregular. The solid red line represents the optimized structure at 200 GPa pressure. Each component direction comprises four peaks corresponding to the directions of the four normal of the tetrahedron, indicating that the tetrahedron after pressure optimization tends to be in the same orientation. As shown in Figure 3d, the distance from each vertex to the plane remains equal, indicating that each N${}_{4}$ molecule is a standard tetrahedral structure.

**Figure 4:**
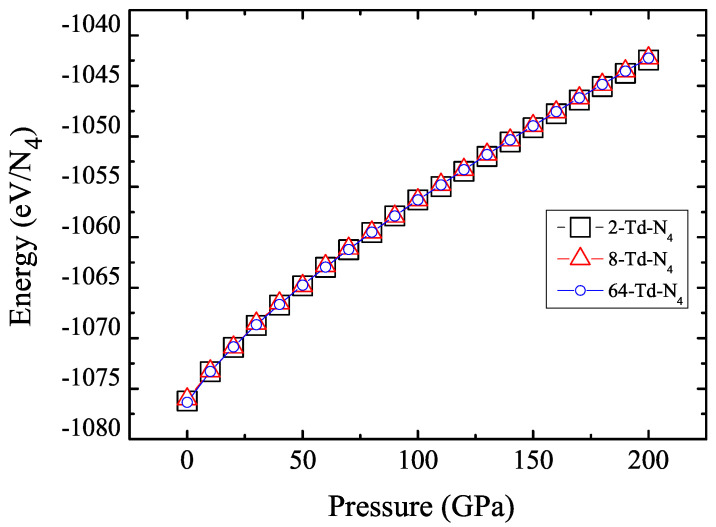
Energy as a function of pressure for the 2-Td-N${}_{4}$, 8-Td-N${}_{4}$ and 64-Td-N${}_{4}$. Black square represents 2-Td-N${}_{4}$, red triangle represents 8-Td-N${}_{4}$, and blue circles represent 64-Td-N${}_{4}$.

**Figure 5:**
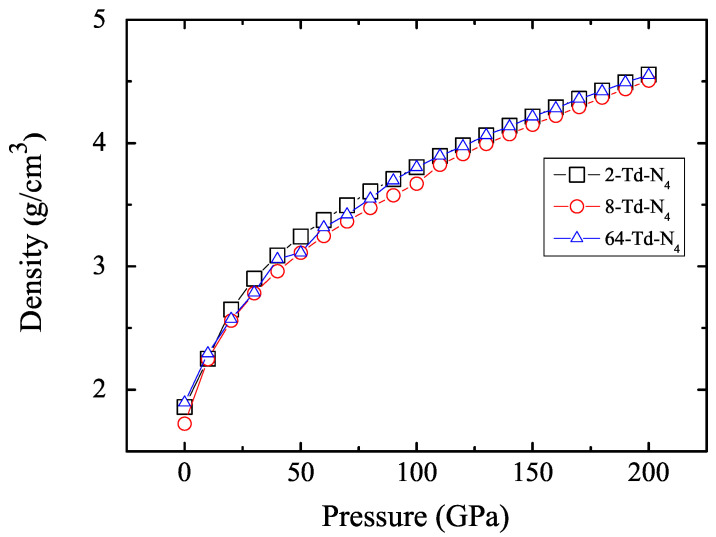
Density as a function of pressure for 2-Td-N${}_{4}$, 8-Td-N${}_{4}$, and 64-Td-N${}_{4}$. Black square represents 2-Td-N${}_{4}$, red circle represents 8-Td-N${}_{4}$, and blue triangle represents 64-Td-N${}_{4}$.

In order to verify the reliability of the results, we compared the average energy per Td-N${}_{4}$ molecule in the three unit cell structures, as shown in Figure 4. Results show that the three structures that we had optimized were convergent. At the same time, we calculated the density of the crystal as a function of pressure. Figure 5 shows that the density of the crystal was about 4.55 g/cm${}^{3}$ when the pressure reached 200 GPa. This is an exciting result because high density is a major factor in HEDMs.

**Figure 6:**
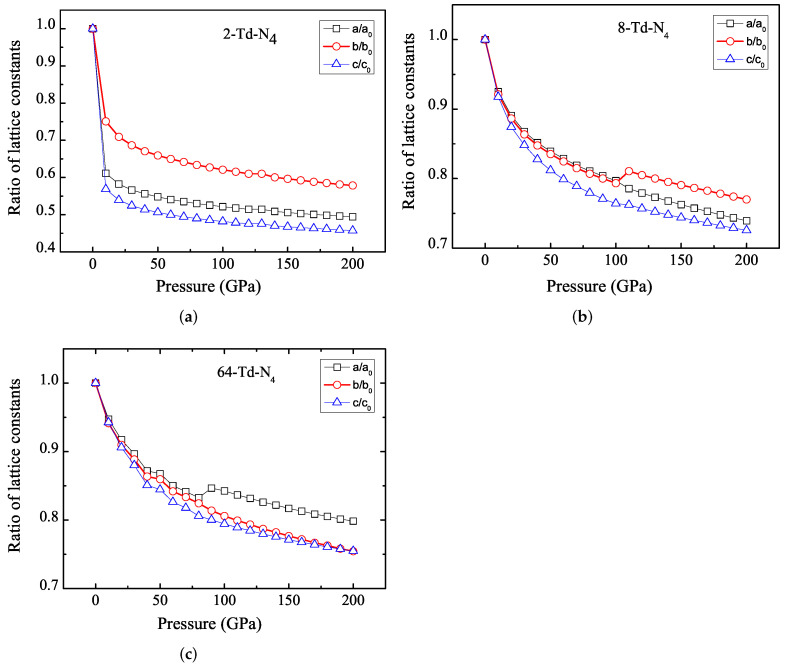
Ratio of lattice constants as a function of pressure for (**a**) 2-Td-N${}_{4}$, (**b**) 8-Td-N${}_{4}$, (**c**) 64-Td-N${}_{4}$.

We studied the pressure-induced changes of lattice constants along three lattice orientations. Figure 6a–c show the change in lattice parameter ratio with pressure for 2-Td-N${}_{4}$, 8-Td-N${}_{4}$ and 64-Td-N${}_{4}$, respectively. The results show that the order of pressure resistance was b-axis > a-axis > c-axis for the 2-Td-N${}_{4}$. 2-Td-N${}_{4}$ had higher compression resistance on the b axis than that on the a and c axes, which reflects the anisotropic molecular interactions in the 2-Td-N${}_{4}$. For 8-Td-N${}_{4}$, a- and b-axis changes were consistent when pressure was less than 100 GPa. As pressure continued to increase, the order of pressure resistance was b-axis > a-axis > c-axis. A- and b-axis changes were consistent for 64-Td-N${}_{4}$ when pressure was less than 80 GPa. As pressure continued to increase, the order of pressure resistance was a-axis > b-axis > c-axis. Our results show that these crystal structures were metastable at high pressure of up to 200 GPa. Insensitivity to external stimulation is still one of the most important requirements for the synthesis of new HEDMs, followed by other characteristics such as high density, higher detonation velocity or pressure, higher environmental compatibility, and high thermal stability.

**Figure 7:**
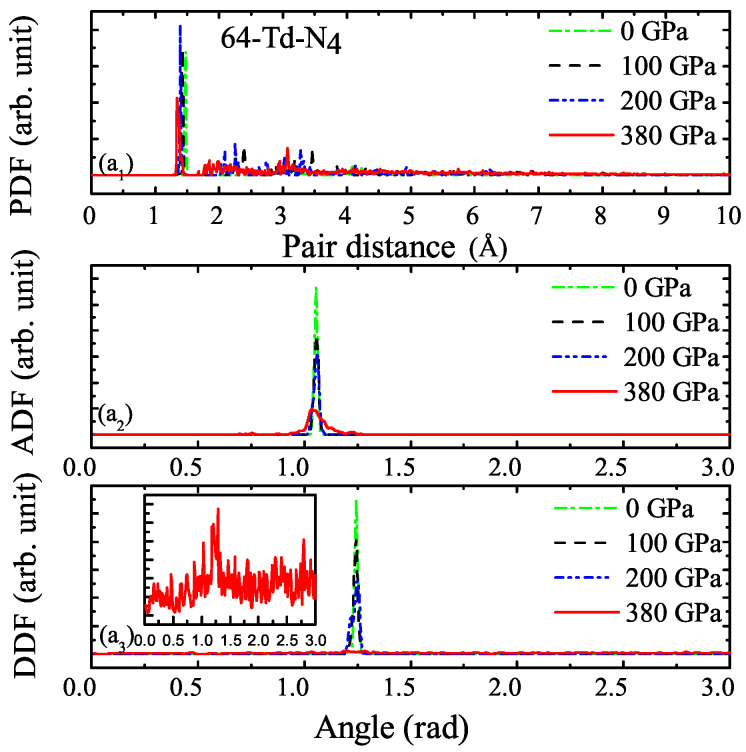
Pair distribution functions for 64-Td-N${}_{4}$ under 0, 100, 200, and 380 GPa. (**a${}_{1}$**) Pair distribution function (PDF). (**a${}_{2}$**) Angle distribution function (ADF). (**a${}_{3}$**) Dihedral distribution function (DDF).

The PDF, ADF, and DDF of 64-Td-N${}_{4}$ under different pressure levels were analyzed, as shown in Figure 7. The main peak represents the bond length and bond angle of the N${}_{4}$ molecule. The main peak was consistent under different pressure levels, which indicates that Td-N${}_{4}$ molecular crystals are only under compressive strain under high pressure, and the essential characteristics of molecular crystal remain unchanged. Figure 7a${}_{1}$ shows that the system exhibited rich structures at r = 2∼5 $\overset{˚}{A}$ under high pressure, which indicates that the pressure compressed the system and reduced the distance between atoms. However, when pressure was 380 GPa, the structure of the system broke, which can be clearly seen from DDF. The illustration in Figure 7a${}_{3}$ is magnified DDF at 380 GPa. Due to the structural fracture of the system, peak values were scattered, and DDF values were small compared with those at other pressure levels.

**Figure 8:**
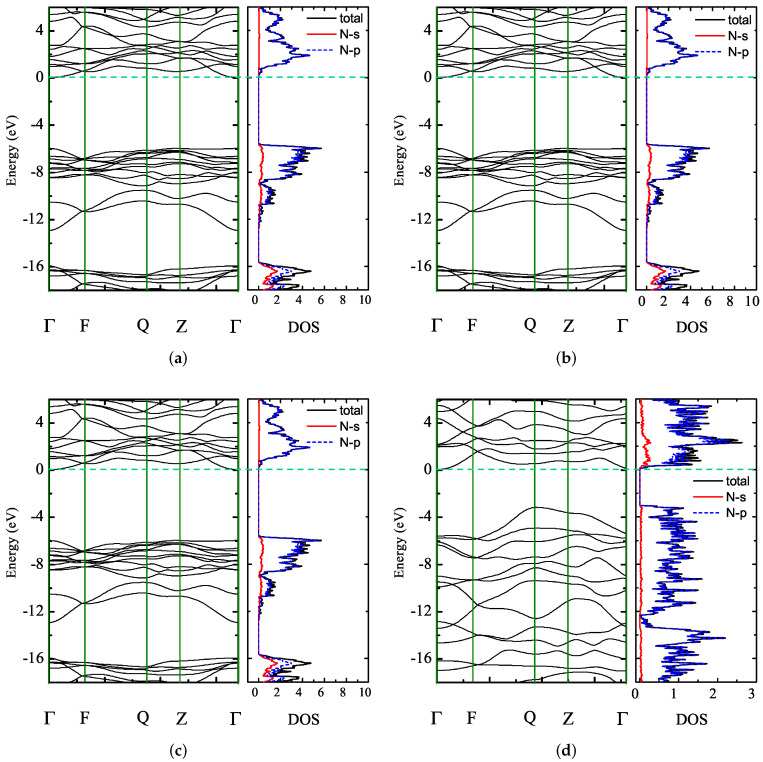
Band structures and DOS of 2-Td-N${}_{4}$: (**a**) 0 GPa, (**b**) 100 GPa, (**c**) 200 GPa, (**d**) 430 GPa. Fermi level E${}_{F}$ was set at zero; horizontal green dashed line denotes the energy positions of the conduction band minimum.

The study of electronic structures is significative to characterize features of high-energy compounds at the molecular and atomic levels. We calculated the band structure and the DOS of the crystal structures under different pressure levels. Taking 2-Td-N${}_{4}$ as an example, Figure 8 shows the band structures and the DOS of 2-Td-N${}_{4}$. Band structures show that the band gap of 2-Td-N${}_{4}$ was about 8.09 eV at zero pressure. As pressure increased, the top of the valence band moved toward the Fermi level, reducing the band gap. As shown in Figure 8b,c, the band gap was 5.96 eV in the range of pressure lower than 430 GPa. Figure 8d shows that the band gap of 2-Td-N${}_{4}$ was obviously reduced to 3.0 eV, which may have been caused by the change in the crystal structure. By observing the geometry, we found that Td-N${}_{4}$ molecules in the crystal structure partially dissociated at 430 GPa.

**Figure 9:**
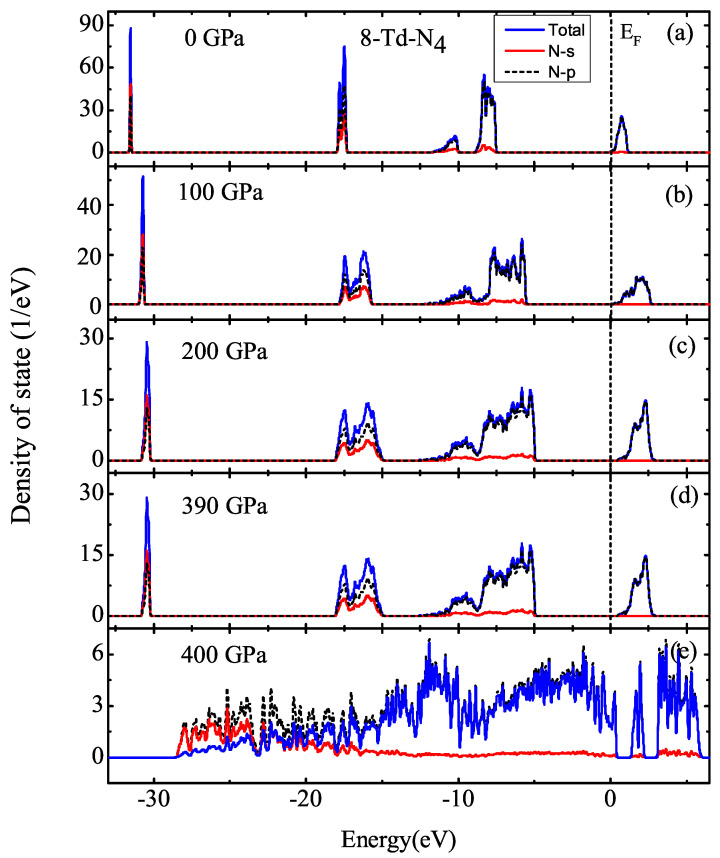
DOS of 8-Td-N${}_{4}$: (**a**) 0 GPa, (**b**) 100 GPa, (**c**) 200 GPa, (**d**) 390 GPa, (**e**) 400 GPa. Fermi level E${}_{F}$ was set to be zero.

**Figure 10:**
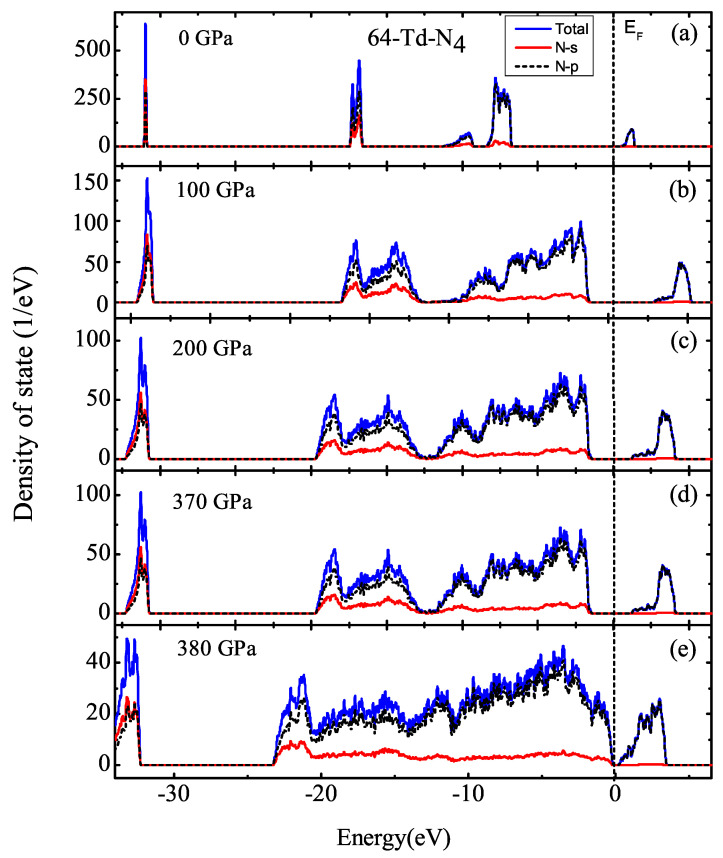
DOS of 64-Td-N${}_{4}$: (**a**) 0 GPa, (**b**) 100 GPa, (**c**) 200 GPa, (**d**) 370 GPa, (**e**) 380 GPa. Fermi level E${}_{F}$ was set at zero.

Figure 8 shows that the band gap was almost constant when the pressure was less than 430 GPa, taking the pressures of 100 and 200 GPa as examples. Combining this with DOS differences for 2-Td-N${}_{4}$ at 430 GPa, the fracture threshold of 2-Td-N${}_{4}$ was 430 GPa. Therefore, high pressure plays an important role in the stability of Td-N${}_{4}$ molecular crystals. Through the calculation of the partial density of states (PDOS), we found that bands were mostly contributed by the p electrons of N atoms. Moreover, the DOS of 8-Td-N${}_{4}$ and 64-Td-N${}_{4}$ was calculated, as shown in Figure 9 and Figure 10. Results show that the fracture thresholds of 8-Td-N${}_{4}$ and 64-Td-N${}_{4}$ were 400 and 380 GPa, respectively. The further increase in pressure caused the partial dissociation of Td-N${}_{4}$, which is consistent with the DDF result in Figure 7.

### 3.2. Properties of Kinetics and Thermodynamics

**Figure 11:**
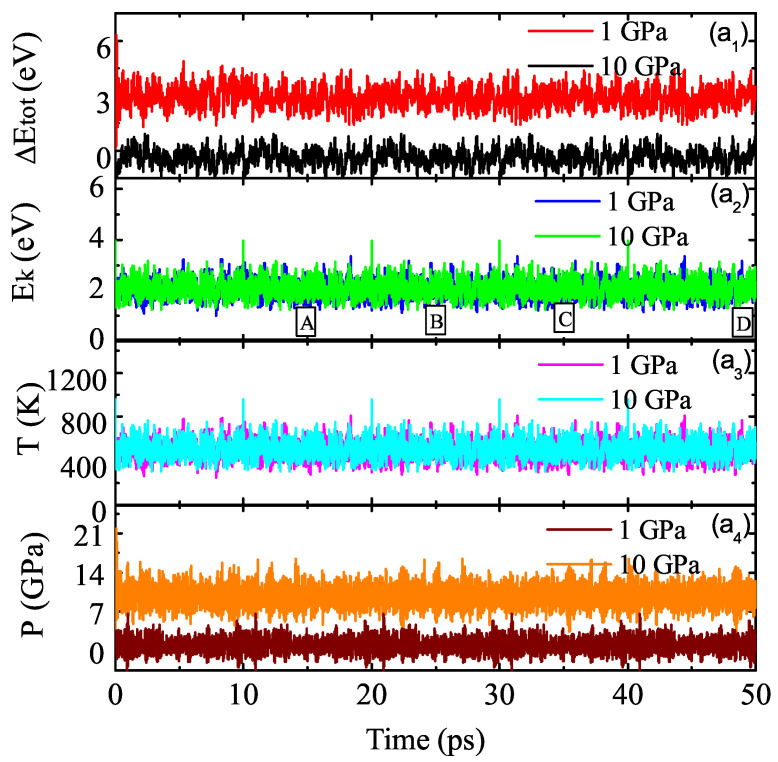
Molecular dynamics simulation was carried out for 8-Td-N${}_{4}$. Figure (**a${}_{1}$**–**a${}_{4}$**) show the total energy (△E${}_{\mathrm{tot}}$(t) = E${}_{\mathrm{tot}}$(t) − E${}_{\mathrm{tot}}$(t${}_{0}$)), kinetic energy (E${}_{k}$), temperature (T) and pressure (P) of the system as a function of time under 1 GPa and 10 GPa, respectively.

To explore thermodynamic stability, a BO-MD simulation was performed under the isothermal–isobaric ensemble. These structures of the molecular crystals of Td-N${}_{4}$ were thermodynamically metastable over the investigated time. Simulations were run for 50 ps at temperature of T = 500 K under pressures of P = 1 and 10 GPa, and the stability of the molecular crystals of Td-N${}_{4}$ was maintained. Here, 8-Td-N${}_{4}$ is taken as an example to study its stability. All MD simulations were performed through the PWmat software package. The structure was relaxed for 50 ps at T = 500 K. Meanwhile, uniform external hydrostatic pressure of 1 and 10 GPa was applied. Figure 11a${}_{1}$–a${}_{4}$ show the total energy (E${}_{\mathrm{tot}}\left(t\right)$), kinetic energy (E${}_{k}$), temperature (T), and pressure (P) of the system as a function of time, respectively. E${}_{\mathrm{tot}}\left(t\right)$, E${}_{k}$ and T tended to be stable throughout the molecular dynamics process.

**Figure 12:**
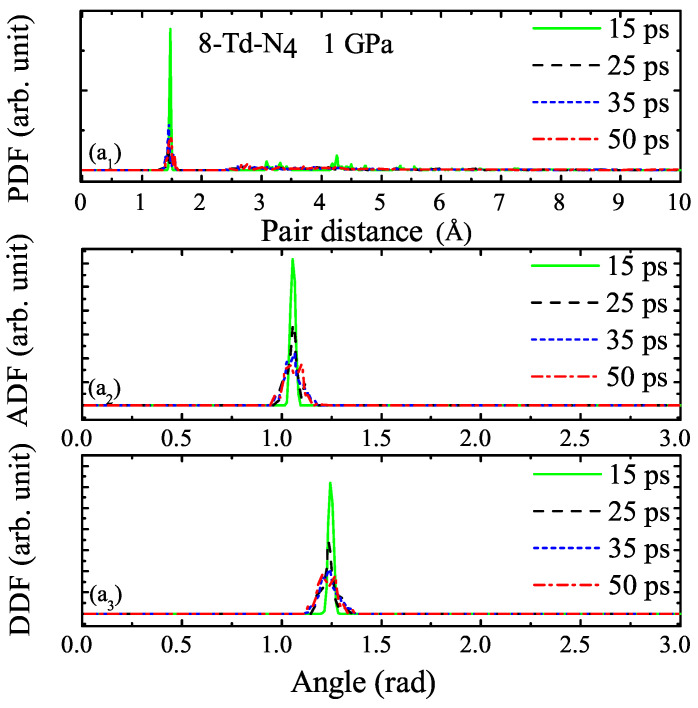
(**a_1_**–**a_3_**) Pair distribution functions at 15, 25, 35, and 50 ps under 1 GPa in the MD simulations.

**Figure 13:**
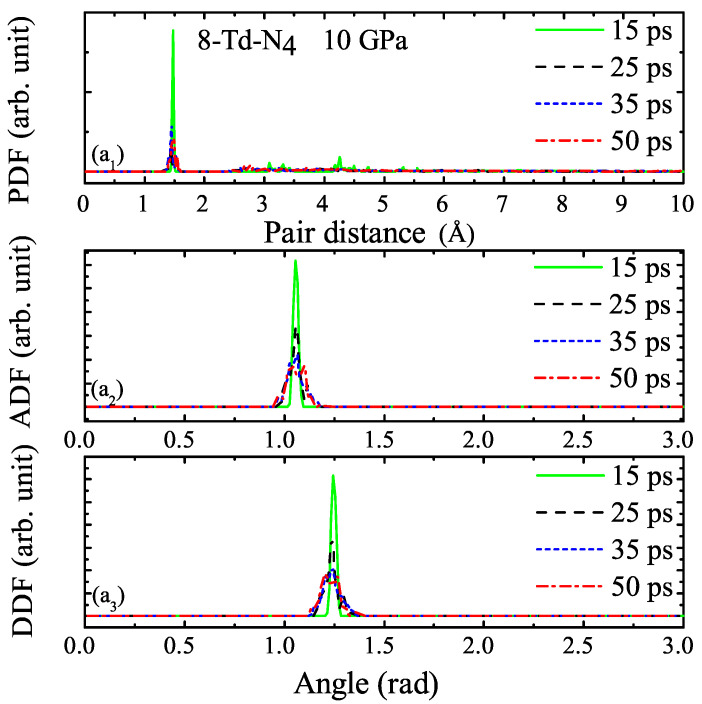
(**a_1_**–**a_3_**) Pair distribution functions at 15, 25, 35, and 50 ps under 10 GPa in the MD simulations.

We selected four different moments, as shown in Figure 11a${}_{2}$A–D, to study corresponding to 15, 25, 35, and 50 ps, respectively. The PDF, ADF, and DDF of 8-Td-N${}_{4}$ under 1 and 10 GPa at each moment are shown in Figure 12 and Figure 13, respectively. Figure 12a${}_{1}$ shows that the pair distribution function was very consistent at each moment in the entire molecular dynamics process. The main peak, which appeared around 1.4 $\overset{˚}{A}$, represents the distance distribution between N–N atoms within the Td-N${}_{4}$ molecule. As shown in Figure 12a${}_{1}$, before the system was thermalized, such as at 1 ps, there were structures during r = 2∼5 $\overset{˚}{A}$. The fully thermalized system after 50 ps had no structure during r = 2∼5 $\overset{˚}{A}$ because of expansion. The peak intensity of the four curves was different, which indicates that intramolecular and intermolecular distances changed. This emphasizes that the pair distribution function of approximately r = 5 $\overset{˚}{A}$ could accurately represent the short-range order between Td-N${}_{4}$ molecules. Similarly, for DDF and ADF, the main peak position was the same at different moments, and peak intensity and peak width were different from the initial moment, which is attributed to the deformation of Td-N${}_{4}$ molecules caused by temperature and pressure throughout the molecular dynamics simulation. However, the crystal was still a metastable structure. Likewise, Figure 13 shows the PDF, ADF, and DDF of 8-Td-N${}_{4}$ under 10 GPa. Distribution functions were basically the same at both pressure levels. The bond length of N–N and the distance between Td-N${}_{4}$ molecules were roughly the same under different pressure levels. The different pressure only changed the lattice parameters, which resulted in a change in cell volume and density. These crystal structures were thus metastable within the pressure range that we studied. In this paper, the data used to calculate the correlation function were as follows: the statistical distance of PDF was r = 2∼5 $\overset{˚}{A}$, the statistical spacing and smearing of PDF and ADF were both 0.01, and the statistical radius of ADF was 2 $\overset{˚}{A}$.

### 3.3. Prediction of Detonation Performance

An important aspect of explosive research is to search for explosives with special detonation, sensitivity, and physical properties. The detonation performance of an explosive determines its application, and detonation velocity and detonation pressure are important parameters to measure detonation performance. Therefore, detonation velocity and detonation pressure can be calculated and used to evaluate the detonation performance of Td-N${}_{4}$. According to the article published by Mortimer J. Kamlet et al. [33,34,35,36], detonation pressures of C–H–N–O explosives could be calculated by means of the simple empirical equation, the Mortimer J. Kamlet and S. J. Jacobs equation (K–J equation) $P\;=\;K\rho_{0}^{2}\varphi$, K = 15.58, $\varphi \;=\;NM^{1/2}Q^{1/2}$; detonation velocities by the equation of $D\;=\;A\varphi^{1/2}\left(1+B\rho_{0}\right)$, A = 1.01, B = 1.30. N is the number of moles of gaseous detonation products per gram of explosive, M is the average weight of these gases, Q is the chemical energy of the detonation reaction ($-△H_{0}$ per gram), and $\rho_{0}$ is the initial density. Here, we predicted the detonation velocity and detonation pressure on the basis of the K–J equation using a program that we had written. This program only requires loading density and an estimate of its enthalpies of formation as input information.

We then wrote a program based on the empirical equation for calculating detonation pressure and detonation velocity. This program only requires the explosive’s composition and loading density and an estimate of its enthalpies of formation as input information. Yingzhe Liu et al. [37] predicted the enthalpy of formation of all-nitrogen materials, including Td-N${}_{4}$. They calculated the enthalpy of formation of Td-N${}_{4}$ on the basis of the atomization reaction, 756.4 kJ/mol, which was 180.8 kcal/mol or 189.1 kJ/mol atom${}^{-1}$. The enthalpy of formation of Td-N${}_{4}$ is slightly lower than that of a **N${}_{6}$** molecular crystal, about 185 kcal/mol [22] but higher than that of bipentazole and N${}_{8}$, about 81.6 and 89.4 kJ/mol atom${}^{-1}$, respectively [38]. The enthalpy of formation of Td-N${}_{4}$ that we used to calculate detonation velocity and detonation pressure was 180.8 kcal/mol.

Another important required parameter in the program is the loading density of explosives. To obtain the density, we used PWmat code to optimize the 8-Td-N${}_{4}$ structure. In the initial structure of 8-Td-N${}_{4}$, molecules were placed in a supercrystal with lattice parameters a = 8.21 $\overset{˚}{A}$, b = 8.36 $\overset{˚}{A}$, c = 8.02 $\overset{˚}{A}$, α = 116${}^{\circ }$, β = 117${}^{\circ }$, γ = 96${}^{\circ }$. For the same lattice parameters, different density values were obtained by applying different pressure levels. As shown in Table 1, as the pressure gradually increased, the density linearly increased; the density even reached 3.67 g/cm${}^{3}$ when the pressure iwass 100 GPa. This is a very significant result because high density is critical to the detonation properties of explosives, such as detonation velocity and detonation pressure. Next, the obtained structure after pressurization was optimized at zero pressure. On the basis of strict convergence criteria, the density of the optimized structure from four structures under zero pressure is 2.09 g/cm${}^{3}$. The van der Waals dispersion effect (VDW = DFT-D2) was considered. There are many theoretical studies on nitrogen cluster, among which the density of Td-N${}_{4}$ predicted in reported studies are 1.752 g/cm${}^{3}$, in [39,40] and 1.81 g/cm${}^{3}$ [41]. Compared with these results, our prediction results are quite exciting, which may have been because we repeatedly optimized the pressure of Td-N${}_{4}$ molecular crystals. This may be understood from the complexity of molecular crystals of N. Next, supercell 64-Td-N${}_{4}$ was simulated at a pressure of 100 GPa on the basis of PWmat code, and the result of the simulation was that the density reached 3.73 g/cm${}^{3}$. At the same time, supercell 64-Td-N${}_{4}$ was optimized at zero pressure. The structure was still metastable after repeated optimization with possible modifications of the cell structure parameters. Supercell 64-Td-N${}_{4}$ optimized at zero pressure can reach a density of 2.20 g/cm${}^{3}$, which is currently the highest among the crystals without exerting pressure in optimization.

The detonation velocity and detonation pressure of explosives could be obtained according to the program when we knew the loading density and enthalpies of formation. For comparison, we also calculated the detonation velocity and the detonation pressure of traditional C–H–N–O explosives 2,4,6-trinitrotoluene (TNT), RDX, 1357-tetranitro-1357-tetrazacyclooctane (HMX), and hexanitrohexaazaisow-urtzitane (CL-20). Results compare well with available data in the literature [42], as shown in Table 2, in which $\rho_{0}$ is the loading density of explosives; ${△}_{f}H^{0}$ is the enthalpy of formation calculated by the atomization energy method; $P_{\mathrm{pro}}$ is the detonation pressure calculated by the program that we had written; $P_{\mathrm{ref}}$ is the detonation pressure in the reference; $D_{\mathrm{pro}}$ is the detonation velocity calculated by our program; $D_{\mathrm{ref}}$ is the detonation velocity in the reference. d-N${}_{4}$ exhibited better overall detonation performance than that of conventional high-energy materials, which shows the potential of new energetic material.

## 4. Conclusions

In this work, on the basis of first-principles Born–Oppenheimer molecular dynamics under constant temperature and pressure, and geometry optimizations under hydrostatic pressure without any constrained parameters, the molecular crystals of Td-N${}_{4}$ were confirmed to be dynamically and thermally metastable. Our analysis shows that, with high detonation performance and high stability, these Td-N${}_{4}$ molecular crystals can indeed be potential candidates as high-energy density explosives. Thus, this should be investigated both theoretically and experimentally in future research.

## Figures and Tables

**Table 1:** Density of the crystal structure consisting of 8 Td-N${}_{4}$ molecules. This structure was optimized by applying external pressure on the basis of a PWmat code.

Pressure (GPa)	Density (g/cm${}^{3}$)	Energy (eV)
30	2.79	−8549.92
50	3.11	−8518.47
80	3.48	−8476.25
100	3.67	−8450.27

**Table 2:** Detonation pressure and detonation velocity calculations for Td-N${}_{4}$ compared with solid C–H–N–Q explosives [42] using the K–J equation.

Explosive	$\rho_{0}{\left(g/{\mathrm{cm}}^{3}\right)\;}^{1}$	${△}_{f}H^{0}{\left(\mathrm{kcal}/\mathrm{mol}\right)\;}^{2}$	$P_{\mathrm{pro}}\;{}^{3}/P_{\mathrm{ref}}\;{}^{4}\left(\mathrm{GPa}\right)$	$D_{\mathrm{pro}}\;{}^{5}/D_{\mathrm{ref}}\;{}^{6}\left(\mathrm{km}/s\right)$
TNT	1.65	−14.17	20.7/19.4	7.01/6.92
RDX	1.80	16.79	34.4/34.9	8.80/8.82
HMX	1.91	17.87	38.5/39.1	9.15/9.15
CL-20	2.04	95.04	44.3/45.9	9.64/9.60
N${}_{4}$	2.09	180.8	74.2	12.27
N${}_{4}$	2.20	180.8	81.0	12.78

^1^$\rho_{0}$ is the loading density of explosives. ^2^ ${△}_{f}H^{0}$ is the enthalpy of formation calculated by the atomization energy method. ^3^ $P_{\mathrm{pro}}$ is the detonation pressure calculated by our program. ^4^ $P_{\mathrm{ref}}$ is the detonation pressure in the reference. ^5^ $D_{\mathrm{pro}}$ is the detonation velocity calculated by the program we wrote. ^6^ $D_{\mathrm{ref}}$ is the detonation velocity in the reference.

## Data Availability

Request to corresponding author of this article.
